# Acute Iatrogenic Agranulocytosis: A Rare and Dire Case of an Adverse Drug Reaction to Be Aware

**DOI:** 10.1155/2020/6125626

**Published:** 2020-11-19

**Authors:** Diogo André, Mónica Caldeira, Fabiana Gouveia, Rafael Nascimento, António Chaves, Maria Braza˜o

**Affiliations:** Hospital Central Do Funchal, Madeira Island, Funchal, Portugal

## Abstract

**Background:**

Iatrogenic agranulocytosis (IA), by nonchemotherapeutic drugs, is a rare adverse event, resulting in a neutrophil count under 0.5 × 10^9^ cells/L with fever or other suggestive signs of infection.

**Methods:**

This paper discusses the possible mechanisms responsible for agranulocytosis induced by nonchemotherapeutic drugs. It also describes three cases as well as potential ways to handle such iatrogenic situations.

**Conclusion:**

Neutropenia under 0.1 × 10^9^ cells/L predispose patients to potentially fatal infections. Empiric broad-spectrum antibiotic and hematopoietic growth factors may be helpful in shortening hospitalization and prevent further infectious complications. Not all drugs associated with IA require frequent hematological monitoring, except medications such as clozapine, ticlopidine, and antithyroids.

## 1. Introduction

Iatrogenic agranulocytosis (IA), induced by nonchemotherapeutic drugs, is a rare adverse effect characterized by the presence of fever or other suggestive signs of infection and an absolute neutrophil count below 0.5 × 10^9^ cells/L [1, 2].

The mortality rate due to IA ranges from 0 to 23%, and avoidance of the responsible agent is vital. However, as a rule, it is difficult to establish this causal relationship between the drug and agranulocytosis [1].

There are two mechanisms by which drugs can induce neutropenia/agranulocytosis [3]. Immune-mediated agranulocytosis results from irreversible binding of the drug metabolite to the neutrophil membrane, resulting in the production of antibodies or stimulation of targeted T cells against the corrupted cell membrane. Just an intermittent exposure is enough, as suggested, with the intake of antithyroids [2, 3]. Direct toxic agranulocytosis results from prolonged exposure of the drug that negatively impacts the bone marrow, culminating in a hypoplastic marrow. It is even admitted that the metabolic detoxification of these drugs results in IA, predetermined by a genetic component [2, 3]. Both mechanisms appear to be mediated by reactive metabolite. The most likely enzyme system responsible is the NADPH oxidase/myeloperoxidase, present in neutrophils and monocytes. However, the exact process responsible for the formation of a chemically active compound still requires further investigation [2, 4].

Its incidence increases with age, as more than half of the cases are reported in individuals older than 60 years and is doubly more frequent in women, reflecting older age multimedication and female longevity [2].

Despite the introduction of new drugs, this incidence is stabilized due to more stringent drug surveillance [2].

Risk factors contributing to IA can be identified, namely, autoimmune diseases (rheumatoid arthritis and captopril), renal insufficiencies under probenecid, and infectious mononucleosis and agranulocytosis secondary to levamisol [3].

There is even evidence of genetic susceptibility to agranulocytosis, namely, the association between HLA-B27/HLA-B38 and clozapine intake as well as the combination of the HLA DRB1*∗*08032 allele in Graves disease with methimazole [3].

The drugs most often associated with agranulocytosis are clozapine, antithyroid, sulfasalazine, and ticlopidine. Most cases of severe neutropenia or agranulocytosis usually occur within the first three months after initiation of the responsible drug [3].

Therefore, a blood count study is vital, as well as educating patients about the most common symptoms of agranulocytosis, as it may contribute to an early diagnosis [5].

Neutropenic AI patients usually manifest as a feverish condition, feeling sick, chills, myalgia/arthralgia, and odynophagia [2, 5].

## 2. Case Series

- Case 1: a 43-year-old woman with recurrent outpatient infections admitted with an oral ulcer, a nodular lesion in the right inguinal region with purulent drainage and thyroid nodules, was treated for a month with methimazole (MM). Her blood count showed 1.8 × 10^3^ *μ*L polymorphonuclear leukocytes (PMNL) with 0.0 × 10^3^ *μ*L neutrophils. Upon completion of broad-spectrum antibiotics, the patient was found to have agranulocytosis due to MM. Normalization of neutrophil counts occurred 11 days after cessation of MM.
- Case 2: a 41-year-old male with schizo-affective disorder receiving clozapine (Cl) had a fever and decreased PMNL count with 0.0 × 10^3^ *μ*L neutrophils. The patient was hospitalized for febrile neutropenia due to neuroleptics, complicated by acute otitis and pneumonia. Following administration of broad-spectrum antibiotics and filgrastim, neutrophil count normalization occurred within 14 days without Cl.
- Case 3: a 61-year-old man with bipolar disorder under Cl was referred for internal medical consultation due to asymptomatic leukopenia with 1.0 × 10^3^ *μ*L neutrophils. Following the suspension of Cl, normalization of the neutrophil count was found 16 days later.

## 3. Discussion

Neutropenia usually resolves within one to three weeks after discontinuation of the drug in question [3].

However, patients with high-risk factors that may dictate a poor prognosis, such as advanced age, renal failure, and septic shock, should be admitted to a hospital unit [2, 3]. Low-risk patients should also be hospitalized, but they can be monitored and treated on an outpatient basis, if possible [2].

A neutrophil count of less than 0.1 × 10^9^ cells/L has been suggested as a poor prognostic criterion and an indication for treatment with growth factors [1]. Although hematopoietic growth factors—granulocyte colony-stimulating factor (G-CSF) and granulocyte macrophage colony-stimulating factor (GM-CSF)—are reserved for particular life-threatening situations, there are no studies with significant associations between the reduction of fatal cases of agranulocytosis and the use of growth factors [1, 2].

Not all drugs, despite a rare association with agranulocytosis, require frequent monitoring. The exception includes medicines such as clozapine, ticlopidine and antithyroid [2].

Clozapine has a cumulative risk of 0.8 to 1.5% per year of determining agranulocytosis. This risk, whose mechanism is likely to be autoimmune in nature, is greatest after 4 to 20 weeks of therapy [4].

Methimazole is one of the drugs often prescribed to control hyperthyroidism. Agranulocytosis, also of an immunological nature, occurs in 0.35% of patients, and it occurs essentially within the first 3 months of therapy [5].

Although, over the last two decades, there has been a decline in cases of agranulocytosis at European level, it is still vital reporting all cases of iatrogenic agranulocytosis, as well as the need for further case-control studies to quantify and assess the individual risk found with the administration of a given drug [2].
